# Chicago Veterinary Society

**Published:** 1896-12

**Authors:** Lawrence Campbell

**Affiliations:** Secretary


Secretary.

### CHICAGO VETERINARY SOCIETY.

On August 7, 1896, a preliminary meeting was held by five Chicago veterinary surgeons to consider the advisability of organizing a society of qualified veterinarians for Chicago and Cook County. On due consideration the idea seemed a good one. One of those present was appointed Secretary, with the request to send notice to all qualified practitioners in Chicago to attend a meeting at the Sherman House, and that the meeting be held one month from that day.

September 9, 1896. On count it was found only eleven practitioners had responded out of a possible eighty-two. They were requested to agitate the question of a society, and each one bring another with him at the next monthly meeting.

October 13, 1896. On count it was found that thirty-three practitioners had presented themselves for membership. A paper was read by Dr. R. G. Walker, Chairman, setting forth the benefits to be derived from a society of qualified men; that such a society might possibly aid the State Association in their fight for legislation; that it might aid veterinarians in securing city positions, such as milk-inspectors and on general dairy-inspection, which properly belong to veterinarians, and also for mutual advancement, as well as allaying petty professional jealousies.

On motion that officers be elected for the Society, the following were elected for one year: Dr. R. G. Walker, President; Dr. O. E. Dyson, First Vice-President; Dr. James Henderson, Second Vice-President; Dr. J. G. Fish, Third Vice-President; Dr. L. Campbell, Secretary; Dr. G. E. McEvers, Treasurer; Drs. Joseph Hughes, Robert Gysel, and L. A. Merillat, Censors. On motion, these gentlemen were elected unanimously.

On motion, the initiation fee was fixed at one dollar, and dues at one dollar per year. Seconded and carried.

Motion being made and seconded, the President appointed three members to draft the by-laws and constitution, same to be ready for the first reading at the next meeting.

A motion that the Society be called the Chicago Veterinary Society was carried.

October 31, 1896. A special meeting was called by the President for the purpose of reading the by-laws and constitution. On count of the members present at roll-call it was found that twenty-two had responded. The by-laws were read, and the meeting adjourned to meet again at the Sherman House on Thursday, November 12th, at 8 P.M., for the re-reading of the by-laws and constitution.

A meeting of the Society was held November 12, 1896, at the Sherman House. On roll-call, fifteen members present. *Seven* new members were admitted at this meeting. The minutes of the last monthly meeting were read and approved by the Society. This being the last reading of the by-laws and constitution, it was moved and seconded that no new members should be allowed to register as charter-members after this meeting.

The by-laws and constitution were then read by the Secretary; after quite a discussion on some of the sections, with some few corrections, a motion was made by Dr. Gysel, seconded by Dr. Henderson, to adopt the by-laws and constitution; carried.

On motion, the meeting adjourned to meet the second Thursday in December, at the Society's rooms, at 8 P.M.

Charter-members: Robert G. Walker, M.D.C.; J. Henderson, M.R.C.V.S.; George E. McEvers, V.S.; James G. Fish, D.V.S.; J. G. Hope, M.D.C.; John W. Foster, M.D.C.; Albert C. Worms, M.D.C.; Ernst Jentzsch, M.D.C.; J. H. Pear, M.D.C.; L. Campbell, D.V.S.; Robert A. Gysel, M.D.C.; James Bond, D.V.S.; P. Quitman, D.V.S.; S. S. Baker, M.D.C.; J. L. Siegroesser, M.D.C.; Charles Roberts, D.V.S.; Joseph D. Tuthill, M.D., V.S.; B. A. Pierce, V.S.; Olof Schwarzkopf, V.S.; Jos. Hughes, M.R.C.V.S.; L. A. Merillat, V.S.; M. H. McKillip, M.D., V.S.; A. H. Baker, V.S., M.D.C.; C. A. White, M.D.C.; Samuel E. Bennett, D.V.M.; J. J. McGrath, M.D.C.; C. G. Nelson, M.D.C.; J. F. Ryan, D.V.S.; James Flemming, V.S.; O. J. Lanigan, D.V.S.; J. F. Black, V.S.; William H. Broderick, M.D.C.; A. E. Flowers, M.D.C.; C. E. Sayre, M.D., D.V.S.; R. H. Tracy, D.V.S.; James McBirney, D.V.S.; A. R. Wake, V.S.; H. W. Hawley, D.V.S.; H. Johnson, D.V.S.; A. E. Rishel, M.D.C.; F. J. Leith, D.V.S.; W. J. Stewart, V.S.; Jacob Berner, D.V.S.; E. L. Quitman, M.D.C., V.S.; B. A. Pierce, V.S.; C. H. Zink, D.V.S.; John Casewell, M.R.C.V.S.; L. Clark, M.D.C.; J. B. Clancy, M.D.C.; James Robertson, D.V.S.; F. H. Davis, M.D.C.—Total, 51.

LAWRENCE CAMPBELL,
Secretary.

